# Mother-to-newborn transmission of mycobacterial L-forms and Vδ2 T-cell response in placentobiome of BCG-vaccinated pregnant women

**DOI:** 10.1038/s41598-017-17644-z

**Published:** 2017-12-12

**Authors:** T. Dimova, A. Terzieva, L. Djerov, V. Dimitrova, A. Nikolov, P. Grozdanov, N. Markova

**Affiliations:** 1 Institute of Biology and Immunology of Reproduction “Acad. K.Bratanov”, Bulgarian Academy of Sciences, Department of Immunobiology of reproduction, Sofia, 1113 Bulgaria; 2 University Obstetrics and Gynecology Hospital “Maichin Dom”, Medical University, Department of Obstetrics and Gynecology, Sofia, 1431 Bulgaria; 3 Institute of Microbiology “Acad. St. Angelov”, Bulgarian Academy of Sciences, Department of infectious microbiology, Sofia, 1113 Bulgaria

## Abstract

The ability of bacteria to exist as a population of self-replicating forms with defective or entirely missing cell wall (L-forms) is an adaptive mechanism for their survival and reproduction under unfavorable conditions. Bacterial mother-to-fetus transfer is a universal phenomenon in the animal kingdom. However, data about vertical transfer of L bacterial forms are extremely scarce. Bacille Calmette-Guérin is an attenuated strain of M. bovis and the only licensed vaccine used for tuberculosis prevention. We already have shown that filterable L-forms of BCG exist freely in the vaccine and are able to reproduce and to form colonies. The present study was focused on the placental microbiome in the context of mother’s BCG vaccination. Here we report an isolation of filterable mycobacterial L-form cultures from gestational tissues and blood of healthy newborns delivered by healthy BCG-vaccinated mothers after normal pregnancy. Of note, vertically transmitted mycobacterial L-forms as a part of placentobiome of the pregnant women didn’t influence the number of resident pathogen-reactive Vδ2 cells. Placenta colonization with mycobacterial L-forms occurs by maternal blood-to-decidua transfer very early in gestation. Together, these data showed that BCG L-forms have the capacity to pass trans-placental barrier and that maternal BCG vaccination affects the placentobiome.

## Introduction

Long-standing paradigm that a healthy pregnancy implies a sterile uterus is already questioned^1^^,^^2^. Recent studies have extended the observations that placenta is colonized by non-pathogenic bacteria (commensals) and have defined placental microbiome (placentobiome) with specific metabolic functions, which differs in term babies and those born prematurely^2^. Health implications from inheritance of such divergent placentobiome and effects on the developing fetus/neonate remain largely unknown. Bacterial transfer from a pregnant mother to the fetus is a universal phenomenon in the animal kingdom. However, data about vertical transfer of atypical (L) bacterial forms are extremely scarce and speculative^3^. The ability of bacteria to exist as a population of self-replicating forms with defective or entirely missing cell wall (L-forms) is an adaptive mechanism for survival and reproduction of bacteria under unfavorable conditions. L-form bacteria have figured out how to successfully live inside the immune cells (macrophages) whose role is to kill bacteria^4,5^. Once inside these cells, they can no longer be detected by the immune system and are able to persist in the body over long periods of time. To date the role of L-form bacteria in infectious diseases has not been fully understood. However, there is evidence that they may be significant in chronic infections^6–8^. We and others have found that filterable L-forms exist freely in the Bacille Calmette-Guérin (BCG) vaccine and are able to reproduce and to form colonies^9,10^. This finding provoked considerable interest in whether BCG L-forms have the capacity to pass trans-placental barrier. Indeed, we were able to isolate mycobacterial L-forms from the blood of newborn babies whose mothers had been BCG-vaccinated 31.2 years ago^11^. BCG is an attenuated strain of *M. bovis* and closely related to *M. tuberculosis* (MbT) as a part of *M. tuberculosis* complex (MTC). Although the ability of BCG to provide an immune protection has been debated since the 1930s, the vaccine was introduced into the Expanded Program of Immunization (EPI) in 1974^12^. Randomized controlled trials have shown efficacies ranging from 0 to 80%^13–15^. Nevertheless the BCG is the only licensed vaccine used for tuberculosis (TB) prevention. More than 3 billion doses have been given since 1948 and BCG coverage is estimated between 26 and 99% (depending on the state)^16,17^. Since 1952, the BCG vaccine has been given routinely and obligatory to babies in Bulgaria at 48 h after birth by intradermal application.

A growing body of data provides convincing evidence that both mycobacterial infection and BCG vaccination induce a specific expansion and phenotype of Vδ2 γδ T cells *in vivo* ^18–21^ and *in vitro* during re-stimulation with mycobacterial lysates and BCG^22^. Moreover, Vδ2 T lymphocytes kill macrophages harboring live MbT through granule-dependent mechanism (granulysin and perforin), resulting in killing of intracellular bacilli and reducing the viability of extracellular MbT^23^. Although Vδ2 T cells typically comprise 5% of total T cells in adult human blood, this population can expand rapidly in response to a wide range of pathogens and is thought to play a key role in human antimicrobial immunity^24–27^. Many bacteria including MbT and BCG produce natural non-peptide low molecular weight phosphorylated metabolites (so called phosphoantigens) which specifically induce Vδ2-cell expansion^19^. Expanded Vδ2 T cells display a range of innate effector functions including rapid secretion of chemokines and cytokines and target cell lysis, as well as contribution to adaptive immunity through B cell help, dendritic cell maturation, and providing of memory function^25^^,^^28–30^. We have recently published data showing that γδ T cells are the first pathogen-reactive immune cells, developed in the fetus. The immune system of mid-gestation fetus contains effector, phosphoantigens-reactive Vδ2 cells with cytotoxic activity and Th1 cytokine profile^31^. During normal pregnancy γδ T cell population at materno-fetal interface is largely comprised of mucosa specific Vδ1 cell subset^32^.

Having in mind all unusual properties of L-forms and their long-lasting persistence *in vivo* ^4,33,34^ and the presence of viable filterable L-form elements in commercial BCG vaccine capable of colonizing the newborns^11^, we aimed to investigate whether the placentobiome of BCG-vaccinated healthy pregnant women contains mycobacterial L-forms able to enter neonate’s circulation and what would be their effect on the amount of placental pathogen-reactive Vδ2 γδ T cells. How early during pregnancy and the possible pathway for L-forms placenta colonization were also examined in this study.

## Results

In total, 79 samples including 27 term placentas, 22 cord blood samples, 13 deciduas, 13 maternal blood samples and 4 trophoblasts were investigated for the presence of mycobacterial L-forms. From 55 samples (20 term placentas, 17 cord blood samples, 6 deciduas, 9 maternal blood samples and 2 trophoblasts) L-forms cultures proven to be of mycobacterial origin were isolated. Mononuclear cell suspensions from 19 term placentas were obtained and subjected to FACS analysis of total T-cell, γδ T-cell and Vδ2-cell numbers.

- MYCOBACTERIAL L-FORMS WERE PRESENT IN TERM PLACENTA AND CORD BLOOD OF NEWBORN BABIES DELIVERED FROM BCG-VACCINATED MOTHERS

### L-forms cultures recovered from placental and cord blood samples

**Figure 1:**
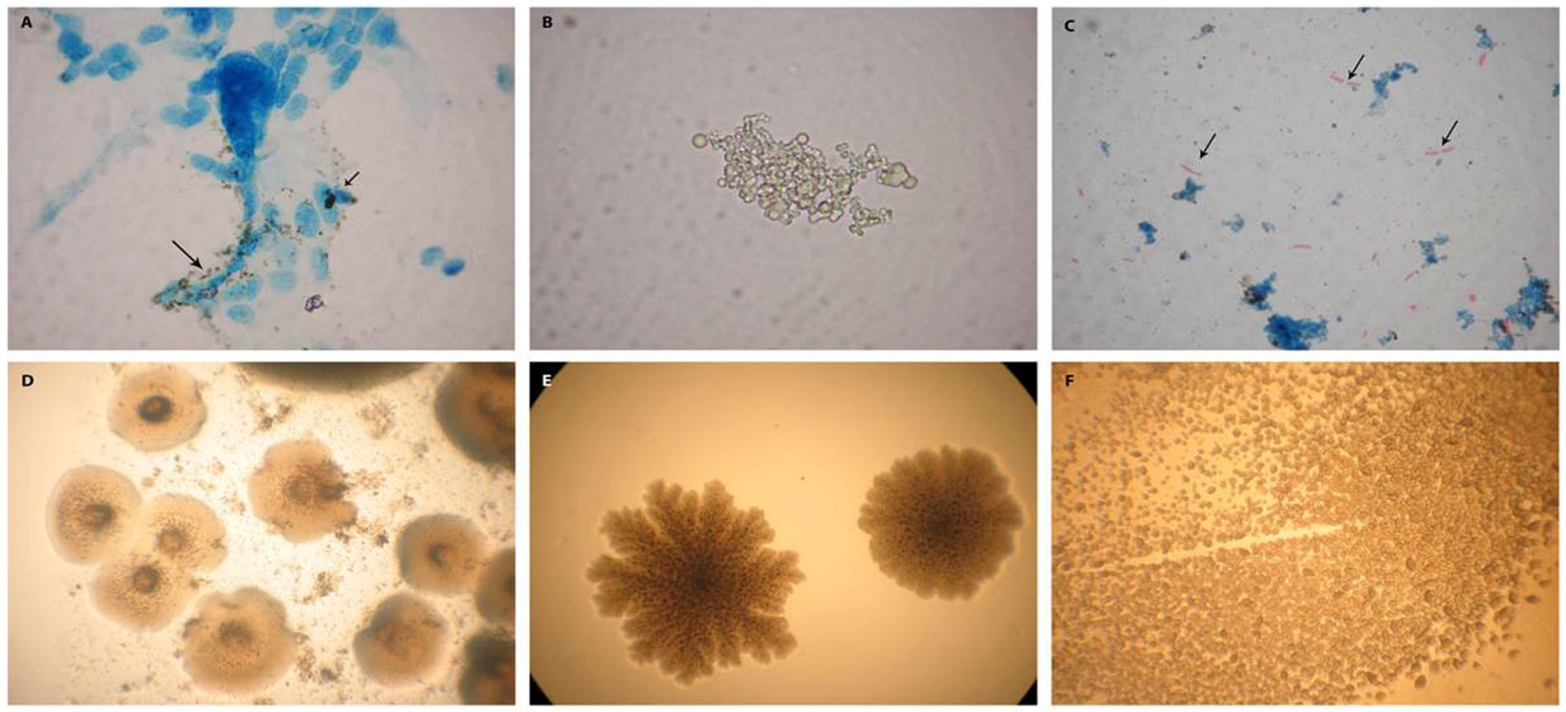
Light microscopy of representative L-form cultures from placental samples: (**A**) Initial phase of L-form development. Arrows point to dark L- form granules associated with placental cells during primary incubation in broth (ZN stained smear, PL-6). (**B**) Development of pure L-form culture in broth after filtration through bacterial filter with 0.2 µm pore size. Native smear –cluster of spherical L-form cells with different size (PL-6). (**C**) ZN stained smear from a broth sub-culture. Arrows point to red stained rods occurring in result to reversion of L-forms to acid fast mycobacteria after further sub-cultivation (PL-6). (**D**) Typical L-form colonies with “fried eggs” shape after sub-cultivation in semisolid medium (PL-6). (**E**) L-type colonies with granular consistence (PL-15). (**F**) L-type tiny colonies (PL-16). Magnification: A, B, C - 1000x; D, E, F- 400x.

**Figure 2:**
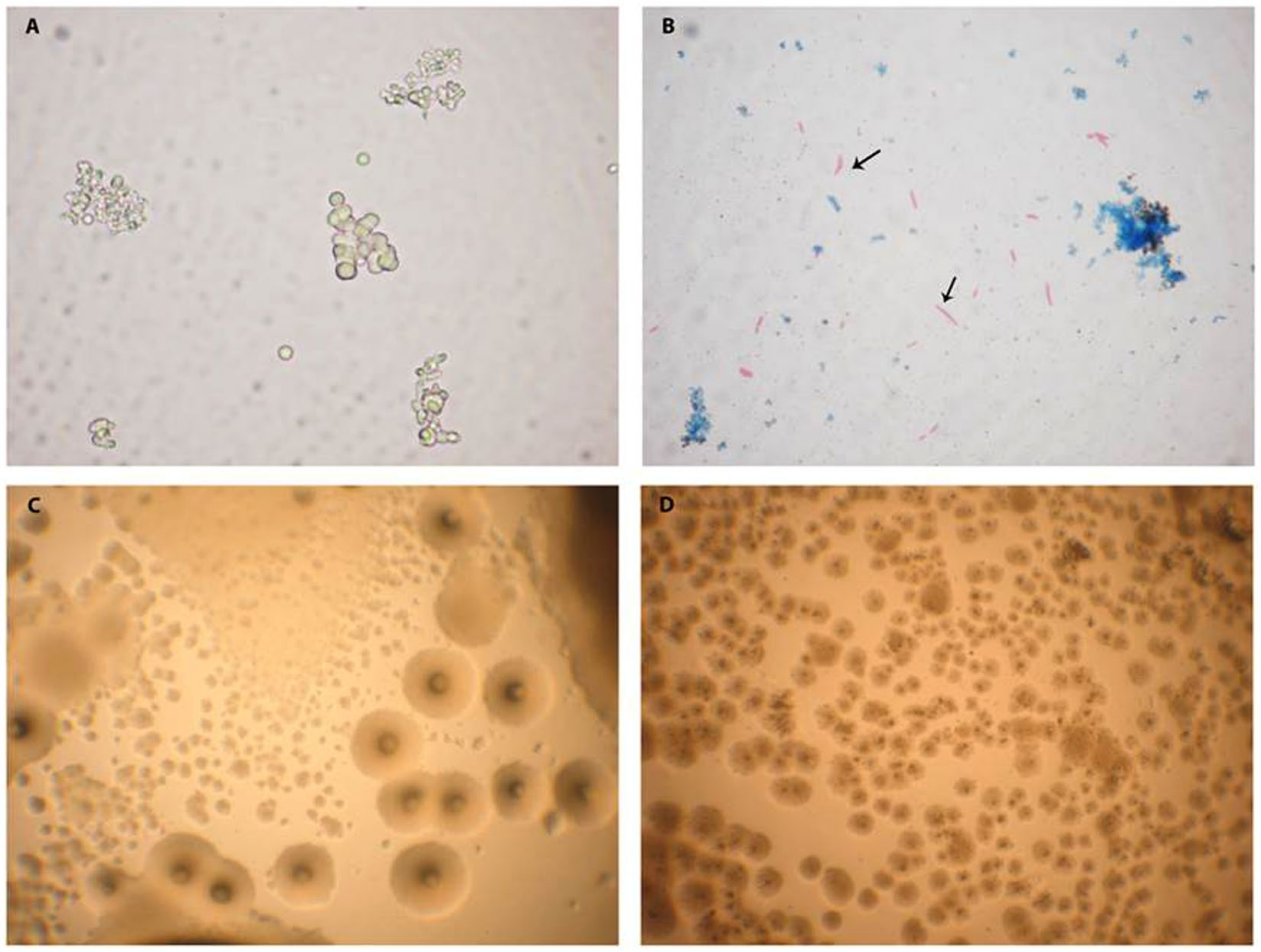
Light microscopy of L-form cultures from cord blood samples. (**A**) Development of pure L-form culture in broth after filtration through 0.2 µm bacterial filter. Clusters of spherical L-form cells with different size - native smear (CB6). (**B**) ZN stained smear from a broth culture. Arrows point to red stained rods, acid fast mycobacteria reverted from L-forms after further sub-cultivation in broth (CB6). (**C**) L-form colonies with “fried eggs” shape after sub-cultivation in semisolid medium. (**D**) Tiny L-form “fried eggs” colonies after sub-cultivation in semisolid medium (CB10). Magnification: A, B - 1000x; C, D- 400x.

For isolation of L-variants here we combine culture studies with careful microscopic observation in order to demonstrate the presence of L-forms in the original samples. The isolated cultures from placenta and cord blood samples showed distinctive growth characteristics of L-form (cell wall deficient, CWD) bacteria. Light microscopy revealed specific for L-forms morphology during the primarily broth and subsequent agar growth phases. Generally, L-forms showed a remarkable heterogeneity in size and shape. It should be noted that dark stained, small L-form granules were observed initially in primary broth cultures, closely associated with stromal placental cells (Fig. 1A). Further broth phase development of pure L-form cultures was presented by native unstained smears. Spherical cells and granular forms of different size in their natural image were seen, demonstrating that cultures were live and replicating (Figs 1B and 2A). Of interest was also the observation in Ziehl–Neelsen (ZN) stained smears of single or few acid-fast bacteria with typical for mycobacteria rod shaped morphology (Figs 1C and 2B). Although the polymorphic CWD cells were prevalent in isolated cultures, the red stained rods seen in about 1% in the observed fields, demonstrated reversion of single L-forms to acid fast mycobacteria (ZN positive). Acid-fastness is a hallmark of typical walled mycobacteria. When the primary broth L-cultures derived from term placenta and cord blood were further sub-cultured in semisolid medium, the appearance of typical granular L-form growth (Fig. 1E, F) and formation of colonies with unique shape of “fried eggs” (Figs 1A and 2C,D) represents a convincing criterion for L-form development.

### Transmission electron microscopy (TEM) of isolated L-forms

**Figure 3:**
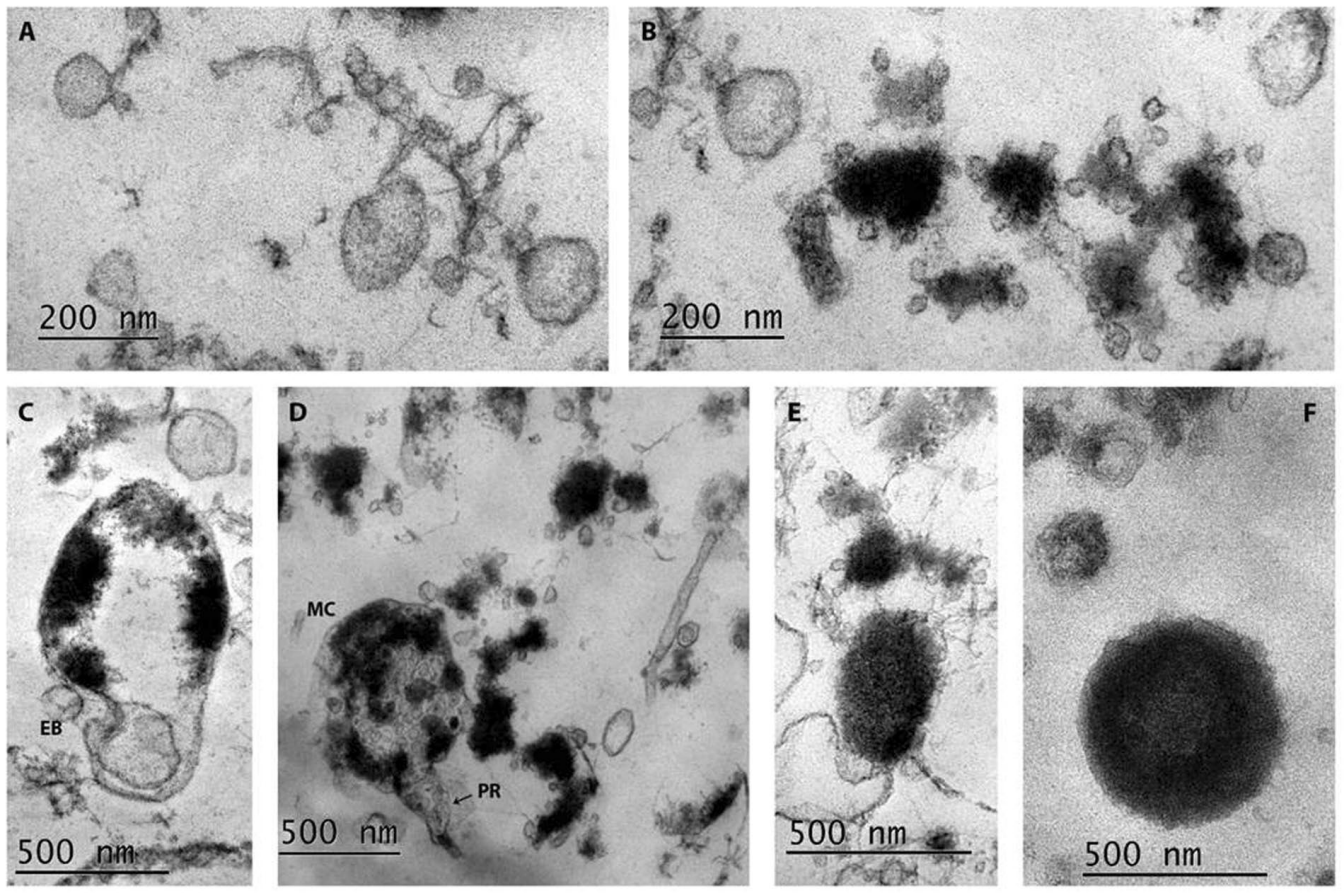
TEM of representative L-form culture, isolated from а placental sample (PL-12) in broth: (**A**) Cluster of small spherical L-bodies with low electron density, some of them with extremely small size - under 100 nm; (**B**) Small electron-dense and shapeless L-bodies associated with multiple vesicular elements; (**C**) Large L-body with condensed cytoplasmic material at the cell periphery. Spherical body inside the cell, invagination of cytoplasmic membrane and releasing of small elementary body are seen (EB); (**D**) Large “mother” cell (MC) containing shapeless and round elementary bodies of varying electron density and size. MC is in process of extrusion of elementary bodies by formation of protrusion (PR). Polymorphic L-form elements are seen around the MC; (**E**,**F**). Spherical or ellipsoid electron dense CWD cells of different size with well-expressed granular consistence.

In correspondence to the light microscopy observations of broth-growing L-form cultures, isolated from placenta and cord blood samples, TEM revealed in depth the polymorphic ultrastructural characteristic of L-form populations and confirmed the absence of any recognizable cell wall-like outer structure. TEM demonstrated the presence of L-bodies with extremely small size of 100 nm and revealed typical for L-forms morphological transformations. Figure 3 displays growth, transformations and replication of bacterial CWD variants within so-called L-form cycle. As seen in Fig. 3A, small spherical L-bodies with low electron density and of different size, some of them extremely small - under 100 nm - were found clustered among fibrillary material. Along with them, small shapeless L-bodies with high electron density associated with multiple vesicular elements were observed (Fig. 3B). In Fig. 3C is presented a large CWD cell with condensed cytoplasmic material at the cell periphery. Probably, these are ribosomes clustered along the cytoplasmic membrane, a phenomenon commonly observed in L-forms. An interesting finding was the visualization of small elementary body inside the cell. Invagination of cytoplasmic membrane and releasing of other small elementary body in the extracellular space demonstrates the process of L-form transformation and mode of unusual propagation. Of special interest was the observation of large “mother” cell (MC) containing shapeless and round elementary bodies, as well vesicles of varying electron density and size (Fig. 3D). Process of protrusion formation and expected subsequent extrusion of elementary bodies demonstrates another mode of L-form transformations and propagations. The generation of intracytoplasmic subunits within the larger maternal cells may be happen by membrane invagination inwards (intracellular budding), occasionally followed by collapse of the maternal cell and mass release of small L-form bodies. It seems that the observed small L-form elements around the MC, as well as these in Fig. 3A and B, confirm this statement and suggest vision for L-cycling processes. The observed larger,”increased in size” spherical or ellipsoid electron dense CWD cells with well-expressed granular consistence (Fig. 3E,F) represent vital, rich of ribosomes, L-forms which are able to replicate within the L-form cycle or revert to walled bacteria.

### Genetic identification of the L-forms recovered from the gestational tissues and cord blood samples by real-time PCR

**Figure 4:**
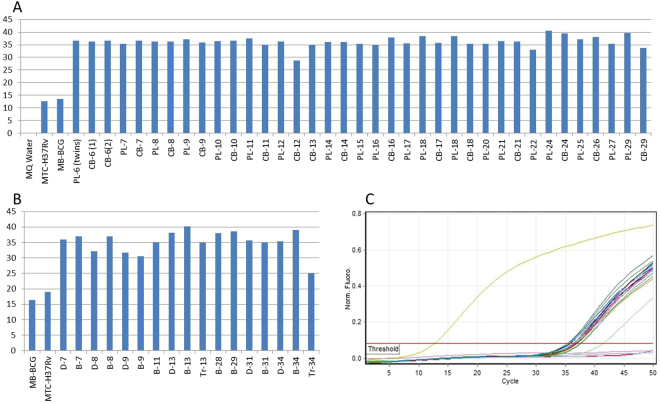
TaqMan PCR for IS1610 detection in filterable L-forms isolated from gestational tissues, cord blood and maternal blood samples. Amplifications were performed with chromosomal DNA from single L-forms colonies. DNA from *M. tuberculosis* H37Rv and *M. bovis* BCG was used as positive controls. MQ water was used instead of DNA template (NTC). (**A**) Graph with raw data (Ct values) of the placenta (PL) and cord blood (CB) samples, positive for mycobacterial L-forms; (**B**) Graph with raw data (Ct values) of the decidua (D) and maternal blood (B) samples, positive for mycobacterial L-forms; (**C**) Report from representative PCR run of DNA from L-forms colonies derived from term placenta and cord blood samples. Tr-trophoblasts.

In order to use more specific methods available today to establish L-forms identity we applied DNA-based assay. To determine whether L-forms isolated in this study were of mycobacterial origin, a TaqMan PCR was performed using specific oligonucleotide probe. The insertion sequence 6110 is a unique conservative marker of mycobacteria belonging to MTC including the vaccinal strain of *M. bovis* BCG. Moreover, IS6110 is present in the genome as multiple copies which significantly increase the sensitivity of PCR amplification^35^. IS6110 PCR revealed that 20 samples (74%) of the investigated individual term placentas and 17 cord blood samples (77%) were colonized with L-forms of mycobacterial origin. Twenty two samples were paired samples (term placenta and cord blood from one and the same neonate) and 85% of these paired samples were positive for mycobacterial L-forms. No IS6110 sequences were generated in non-template controls. The PCR data are shown in Fig. 4. All positive samples were subjected to PCR at least twice in some cases thrice to be sure that L-form-positive samples are really positive. Although we used probe-specific PCR method we considered as negative all samples showing Ct > 40 cycles.

**Figure 5:**
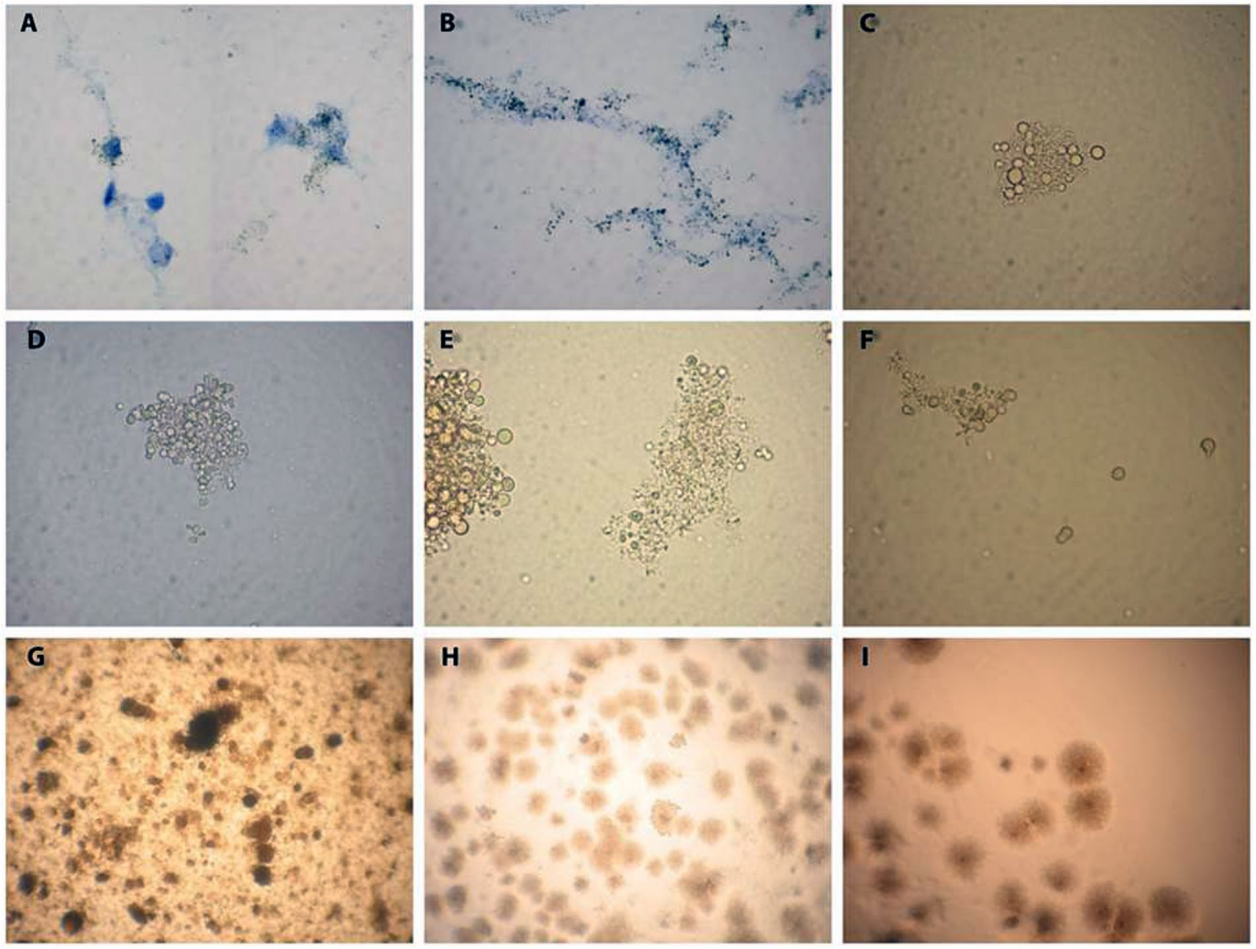
Light microscopy of L-form cultures from decidua (D) and maternal blood samples (B). (**A**) Initial development of L- forms (dark granules) associated with decidua cells in broth (D34). (**B**) Dark L-form granules along decidua cell debris in broth (D31). (**C**) Native smear from a pure broth L-form culture, developed after filtration through bacterial filter with 0.2 µm pore size. Cluster of spherical cells with different size (D31). (**D**–**F)**. Native smears with spherical and granular L-form cells in broth L-form cultures from maternal blood samples B9, B31 and B34 respectively; (**G**–**I)** (L)-form growths after sub-cultivation in semisolid medium. Granular L-form growth of B9 (G), typical L-form “fried eggs” colonies of D8 (**H**) and D9 (**I**). Magnification: A, B, C, D, F- 1000x; G, H, I - 400x.
- THE PRESENCE OF MYCOBACTERIAL L-FORMS DID NOT INFLUENCE THE AMOUNT OF RESIDENT PATHOGEN-REACTIVE Vdelta2 CELLS

- MATERNAL BLOOD-TO-DECIDUA TRANSFER OF MYCOBACTERIAL L-FORMS OCCURED EARLY IN GESTATION (1^ST^ TRIMESTER)
  Next questions we would like to answer were how early in the course of pregnancy the placenta became colonized with mycobacterial L-forms and whether the maternal decidua takes part in L-forms transmission. For this purpose we investigated 13 paired samples maternal blood and decidua derived from women in early pregnancy (6–12 gestational weeks, gw). In 6 out of 13 paired samples (46%) both maternal blood and decidua were colonized with L-forms genetically identified as mycobacterial L-forms (Fig. 4B). Four out of 13 paired samples (31%) were negative for mycobacterial L-variants. In the remaining 3 paired samples, however, we detected mycobacterial L-forms only in the blood of pregnant women but not into decidual tissue (Fig. 4B). L-form cultures isolated from maternal blood and early decidua samples resembled those isolated from term placenta and cord blood samples, showing the same morphological and growth characteristics. As seen in Fig. 5A and B, dark stained L- form granules were initially observed and closely associated with decidual cells in primary broth cultures. Native smears of L-form cultures demonstrated a variety of vital spherical cells and granular elements of different size (Fig. 5B,C,D,E). The observed granular growth and “fried eggs” colonies after sub-cultivation of the isolates in semisolid medium were typical for L-forms (Fig. 5G,H,I). Two of the positive paired samples (6 gw and 11 gw) were completed with positive trophoblasts as well (13 D-B-Tr and 34 D-B-Tr, Fig. 4B).

**Figure 6:**
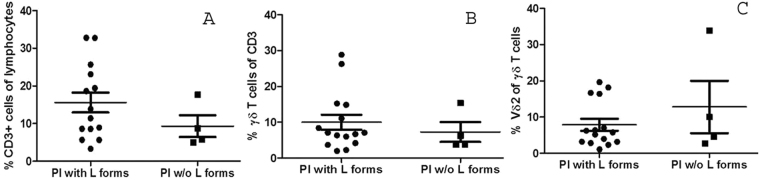
The presence of mycobacterial L-forms in the placentobiome of BCG-vaccinated pregnant women has no an impact on the numbers of resident T cells, γδ T cells and phospho-reactive Vδ2 cells. Pl – placenta.

Since Vδ2 cells are key early sensors of mycobacterial infection and are specifically activated and expanded by MbT- and BCG-released phosphoantigens^36^ we further aimed to find out whether the colonization of the placentobiome with mycobacterial L-forms might drive an expansion of resident pathogen-reactive Vδ2 T cells *in vivo*. Thus, we compared the number of Vδ2 cells in placental samples positive and negative for mycobacterial L-forms. Our results showed that there is no difference in the amount of resident Vδ2 cells between both groups. In addition, the numbers of total T cells and γδ T cells were comparable as well (Fig. 6).

## Discussion

The present study is focused on the placental microbiome - new and hot topic of research - in the context of mother’s BCG vaccination. Here we report for the first time to our knowledge an isolation of mycobacterial L-form cultures from gestational tissues of healthy newborns delivered by healthy BCG-vaccinated mothers after normal pregnancy. Since BCG vaccination is currently recommended in 156 countries^37^ and mandatory in 64 of them^38^, this study opens the question whether the vaccination in the childhood of the woman has an impact on her placental microbiome during pregnancy. Although the effectiveness of the BCG vaccine has been debated for decades, the possible trans-placental transfer of BCG L-forms as a phenomenon affecting fetal and/or neonatal immune system is not described so far.

Our study clearly demonstrates four major findings: 1) presence of mycobacterial L-forms in the placenta and cord blood of healthy newborn babies delivered from healthy BCG-vaccinated mothers; 2) vertically transmitted mycobacterial L-forms originated from the maternal BCG vaccine have a capacity to revert back to walled mycobacteria; 3) placenta colonization with mycobacterial L-forms occurs by maternal blood-to-decidua transfer very early in gestation (1^st^ trimester) and 4) mycobacterial L-forms as a part of placentobiome of BCG-vaccinated pregnant women did not influence the number of placental pathogen-reactive Vδ2 cells.

L-forms differ fundamentally from normal state of bacteria. They exist without cell walls, assume mostly spherical shapes and are capable of multiplying through unusual modes, such as irregular binary fission, budding, protrusion-extrusion of elementary bodies and granules from large bodies, multiple divisions with intracellular fragmentation of cytoplasm, or combination of all types^39^. Recent experiments by Loessner’s group have shown that L-forms are an independent form of life that can multiply indefinitely as the authors said “an alternative form of bacterial life”^40^. Beyond doubt, our morphological examination of isolated L-form cultures and especially TEM observation on placental L-forms, point to an amazing cell division mechanism in which new L-form vesicles are formed (by membrane constriction i.e. invagination) and develop within large ‘mother’ cell. When the mother cells enlarge enough a release of daughter cells (new L-forms) occurs (see Fig. 3). Our data fits well with recently published time-lapse confocal laser scanning microscopy data of the growth and multiplication of GFP-labelled L-forms showing different steps of L-form propagation as a novel model for the growth and division of L-forms of *Listeria*, *B. subtilis*, *E. coli*, *S. aureus*, and *C. glutamicum* ^41,42^. In line with other researchers and our previous studies, we found here that L-forms in gestational tissues and blood exist as unusual morphological units, such as large and elementary bodies, vesicles, granules, filterable forms, and display typical L-form growth. Although polymorphic, L-forms usually form colonies with unique shape of “fried eggs”, which are accepted by many authors as a convincing criterion for L-form growth^7,33,39,43,44^. Crucially, IS6110 Real Time PCR assay confirmed that L-form isolates from gestational tissues and maternal/cord blood are mostly of mycobacterial origin. Our recent publications showed that mycobacterial L-forms persist in blood of BCG-vaccinated people and that filterable, self-replicating bodies with virus-like size of 100 nm are able to cross the materno-fetal barrier, and by falling in fetus blood circulation to colonize the newborn^10,11^. Keeping in mind that L-form cultures were isolated after filtration of broths, inoculated with gestational tissue, maternal blood or cord blood samples, we suggest that very small L-bodies (filterable forms) are able to pass through pores with 0.2 µm size, germinate again and to launch a new life L-cycle. The filterable forms have been considered as minimal reproductive cells, which can be formed from large L-bodies in all possible ways. It is believed that such filterable bodies contain a bacterial genome and minimal metabolic capability sufficient to initiate reproduction^7,39,45^. One of characteristics that distinguish L-forms from walled bacteria is loss of peptidoglycans (PG), respectively PG -associated molecules and their functions^46,47^. Of interest, some authors accept that electron-dense spots found in L-form membrane regions, observed by TEM also in our study, are accumulated PG precursor molecules made in the cell cytoplasm which normally would be inserted into the existing PG meshwork outside of the membrane^48^. In general, L-forms are thought to be unstable and genetically identical to their parent strain. They retain the ability to revert back to the classical walled form, suggesting that the ability to rebuild a cell wall *de novo* is also a common property of bacteria^49,50^. Indeed, isolated filterable L-forms in our study were unstable and reversible and we successfully visualized CDW bacteria together with their walled counterparts - ZN-positive typical rod-shaped mycobacterial bacilli in the cultures of placental and cord blood isolates.

Demonstration that BCG bacilli can convert to CWD forms inside resting or pre- activated macrophages *in vivo* suggests that this phenomenon could significantly enhance BCG survival and persistence ability^4,33^. Moreover, the well-packed BCG L-forms could be released to the extracellular space probably exploiting apoptotic-like pathway for subsequent rounds of new entry and uptake by macrophages^4^. Thus, the L-form state of *M. bovis* could be regarded as an important factor for the long-term survival strategy of this pathogen. Actually, little is known how long *M. bovis* BCG as a live strain can survive in the vaccinated persons. There are reports about detection and isolation of BCG bacilli from patients with AIDS many years after their vaccination^51–53^. Recently published data showed that conversion of bacteria to L-forms, may often result in chronic infections, since L-forms remain slumbered for long periods in the tissues becoming sequestered in protective regions of the body^7^. Placenta is considered as such a protective and immune privileged organ, where an immune tolerance must be established in the course of normal pregnancy. The placenta forms a barrier between mother and fetus and in this respect it provides immunologic control on the transfer of gas, nutrients, pathogens and antigens from mother to fetus. In human invasive implantation and hemochorial type of placenta, placental trophoblast cells come in close contact with the maternal tissues forming two materno-fetal interfaces. One materno-fetal interface is that wherein the maternal decidua contacts with the interstitial placental cytotrophoblasts. The other one is formed between the placental chorionic villi (covered by syncytiotrophoblasts) and the maternal blood and increases in volume as pregnancy progresses. We still know very little about the identity and number of microbes that traverse the placenta, whether they persist in the infant or whether their presence has short- or long term health consequences. Much of the current knowledge about placental barrier comes from the understanding of its impermeability to many large molecules and circulating pathogens^54^. Different pathogens can infect and cross the placenta include viruses (e.g. Cytomegalovirus, HIV), protozoa (e.g. Toxoplasma, Trypanosoma, Leishmania, Plasmodium), and bacteria (e.g. Treponema, Brucella, Listeria)^55,56^. Among macroparasites, some helminths have also been observed to cross the placental barrier (e.g. Toxocora^57,58^ and Trichinella^59^). Silica nanoparticles smaller than 300 nm have been shown to penetrate the placental barrier in mice^60^. Nano-sized vesicles of 30–100 nm in diameter (exosomes) and their trafficking within the placental micro-environment have been recognized to play a role in mediating embryo-maternal interactions^61,62^. It has been shown that exosomes, released by BCG-infected macrophages contain mycobacterial components^62^. Summing up the accounted data above and dual materno-fetal contact we could suggest couple of routes for vertical transmission of mycobacterial L-forms such as hematogenous transfer across the placenta by direct infection of trophoblasts or through intercellular communication, placental transfer of the infected immune cells from the maternal blood stream and/or transfer of mycobacterial L-forms from infected maternal decidua. The later pathway is supported by our finding that in half of the paired maternal blood-decidua samples, the decidua was already colonized with mycobacterial L-variants. Moreover, two of the positive paired samples were completed with positive trophoblasts as well showing that L-forms were transmitted to early placenta (trophoblasts) through maternal decidua. Although we succeeded to isolate mycobacterial L-forms from gestational tissues as early as 6 weeks of pregnancy would be interesting to screen more triple samples maternal blood-decidua-trophoblasts in order to find out convincing data about direct and/or mediated by the maternal decidua L-forms infection of trophoblasts.

In agreement with previous studies we found that in human term placenta γδ T cells account for 5–10% of CD3+ lymphocytes^63^. About 10% of the resident γδ T cells are phosphoreactive Vδ2 cells, which is in line with data showing that the majority of the placental γδ T cells are Vd1^32^. Our results demonstrate that the presence of mycobacterial L-forms in the placentobiome of BCG-vaccinated pregnant women had no impact on the number of placental Vδ2 cells. We could not exclude, however, a possible reflection on their phenotype. In normal state mycobacteria four different phosphoantigens termed TUBag1 to TUBag4 have been isolated and identified from the mycobacterial wall^64^ suggesting that the maintenance of specific Vδ2 immunity necessarily needs BCG bacilli to produce these immunogenic cell wall-associated compounds^65^. When bacteria shed their cell wall, they might also lose factors contributing to their specific ‘pathogen-associated molecular pattern’ important for recognition of the invader by the (innate) immune system of the hosts^66–68^. Data from experimental infections with L-forms demonstrated their atypical interactions with phagocyte cells and incomplete and ineffective phagocytosis^69–72^. As the bacterial cell wall is highly immunogenic, several groups have tried to elucidate whether or not CWD bacteria evoke an immune response and, if so, how this response differs from that triggered by bacteria harboring a cell wall (reviewed in)^73^.

Some authors have found that the L-forms were pathogenic only when reverted in the host to bacterial forms^74^. As discussed by Errington *et al*. these reports show controversial results and most of the work is difficult to interpret in the light of our modern understanding of immune mechanisms, particularly innate immune responses^8^. Thus, although not proven yet it is reasonable to assume that when BCG bacilli transform in CWD forms, probably they cease and/or decrease their immunogenicity but still could provide a reservoir hidden from the immune system and resistant to treatment with cell-wall-specific antibiotics.

Despite large amounts of literature published on L-forms, atypical bacterial forms have been neglected by clinicians for very long time because of difficulty to identify and prove them. However, a lot of papers and reviews^44^^,^^6^^,^^7^ support the concept that L-forms can be induced *in vivo*, can persist there for a significant span of time and can be the cause for latent, chronic and relapsing/recurrent infections, as well as for diseases of unknown infectious-allergic or autoimmune origin. Combination of modern imaging techniques and molecular approaches, such as generation of stable L-form lines and fluorescently stained L-form cells, an array-based transcriptomics of parent and L-form cells have been applied in order to better understand L-form conversion and the molecular and genetic changes accompanying this unusual transition^40^^,^^8,42^. The change from the normal form to the L-form is accompanied by drastic changes in cell metabolism and gene activity, suggesting that L-forms can adapt to their cell wall deficiency by adjusting expression levels of genes important for survival and adaptation to this unusual life style^41^^,^^75^.

In summary, this study identifies novel data about mycobacterial L-forms colonization of gestational tissues (placenta, decudua) and cord blood of healthy newborns delivered by healthy BCG-vaccinated pregnant women and provides the first formal demonstration that maternal BCG vaccination affects the placentobiome. Work on L-forms in cord blood provides food for thought in terms of the safety of the use of cord blood stem cells for bone marrow transplantation. Our research is ongoing to define how maternal decidua mediates the process of mycobacterial L-forms colonization of placenta. With the results here we hope to stimulate a research on L-forms in the placentobiome and their short- and long-term effects on the immunity of the fetus/newborn as well as the relationship of these cryptic organisms with autoimmune diseases in adulthood.

## Materials and Methods

### Study population

Two groups of healthy pregnant women were enrolled in this study as follows: 1) pregnant women in early pregnancy, directed to elective pregnancy termination (6–12 gw, n = 16) and 2) women who delivered at term (38–40 gw, n = 27). None of the women had history of acute, chronic or congenital infections. All pregnant women met the following criteria: vaccinated with BCG (at birth), without history of exposure to TB, negative for TB by PPD skin test (Mantoux test), with normal pregnancy and delivery. All the newborn were clinically healthy. Birth weight of neonates was between 2930 and 3710 g, and the length – between 47 and 52 cm. This study was carried out in accordance with the Declaration of Helsinki and was approved by Human Research Ethics Committee at the University Obstetrics and Gynecology Hospital “Maichin Dom” and the Medical University. Written informed consent was taken from all subjects for the use of blood and tissue samples for research purposes and all specimens were handled and anonymized in compliance with national guidelines.

### Samples from gestational tissues and from blood/cord blood

Cord blood and placenta samples were taken from pregnant women (38–40 gw) after delivery and maternal blood, decidua and trophoblasts samples were taken from pregnant women (6–11 gw) at pregnancy termination. All primary specimens were collected within 1 hour of abortion/delivery under clean and sterile conditions. After collection the tissue specimens were placed on dry ice in sterile containers with PBS and transported to the laboratory to be processed immediately for different analyses. Cord blood samples were obtained from umbilical vein of newborns. Blood samples were aseptically collected using standard technique with a needle that was connected to K2E-EDTA Vacutainer tubes (BD Vacutainer, Plymouth, UK) and a cord blood flowed through the needle into the tube.

### Isolation of L-form cultures

Pieces of approximately 1 to 2 g from placental/decidual samples were homogenized with sterile distillated water and were used for isolation of L-form cultures. Isolation of L-form cultures from placental, decidual, cord blood and maternal blood samples was performed according to the protocol described in our previous study^10^. In brief, blood lysis was done with sterile distilled water at strictly fixed v/v ratio and after 30 min exposure to room temperature. The aliquots from lysed blood samples and from placenta/decidua tissue homogenates were inoculated in tubes with Tryptic Soy Broth (TSB, Becton Dickinson), which were then filtered through a bacterial filter with 0.2 µm size of pores and incubated at 37 °C for 72 hours. Then, strictly fixed aliquots from primary broth were sub-cultured again in broth and parallel plated with special technique on Petri dishes with semisolid medium. Both broth and agar cultures were incubated at 37 °C for one week. The semisolid medium was prepared from TSB solidified with 0.8% (w/v) Agar (Fluca). The cultures were examined macro- and microscopically for appearance of growth. Direct light microscopic observations of cultures were combined with ZN stained preparations.

### Transmission electron microscopy

Observations of broth L-form cultures from placental and decidual samples were performed by electron microscopy. A depot from broth L-form culture was harvested by centrifugation at 3000 rpm for 20 min. After that, the depot was fixed with 4% (v/v) glutaraldehyde in 0.1 M cacodylate buffer with 4.5% w/v sucrose, pH 7.2 and post-fixed in 1% (w/v) osmium tetroxide in the same buffer at room temperature for 2 h and dehydrated in serial ascending ethanol concentrations. After dehydration in ethanol and propylene- oxide series, cell pellets were embedded in epoxy resin Epon-Araldite (Serva, Heidelberg, Germany). Resin blocks polymerized at 56 °C for 48 h. Ultrathin cell sections were made with crystal glass knives on a Reichert-Jung Ultracut Microtome and were stained with 5% (w/v) uranyl acetate in 70% (v/v) methanol and 0.4% (w/v) lead citrate. Observations were made with electron microscope JEOL JEM -1011 SAP10 (Japan) at 40–100 kV.

### Real Time PCR detection of specific for MTB complex IS6110 in L-form cultures

Depots from broth L-form cultures were used for genetic testing. Several precautions were taken to avoid contamination during the extraction procedure and in the PCR reactions. The DNA extraction, PCR and post-PCR analyses were conducted in separate laminar flow biosafety cabinet and rooms. Sterile aerosol protection filter tips were used to avoid cross-contamination. Two extraction blanks were always included in the same procedure and an additional PCR blank was included in each PCR reaction, containing no DNA template. Chromosomal DNA was isolated as described by Embden *et al*.^76^ Real Time PCR mixtures containing a final concentration of 1X PCR buffer (Tris.Cl; KCl; (NH_4_)2SO_4_; 15 mM MgCl_2_, pH 8, 7, Qiagen), 2.5 mM MgCl_2_, 0, 2 mM of each dNTPs (dNTP Mix, Qiagen), 1,75 U HotStarTaq DNA polymerase (Qiagen), the target specific primers and probes, were used at a final concentration of 0.5 mM, finally 5 ml of DNA template was added. The reaction mixture was performed in a final volume of 30 ml. The primers and the probe sequence were selected from a region of the IS6110: Primers IS6110 D-1 (50-ACCTGAAAGACGTTATCCACCAT-30) and IS6110 D-2 (50-CGGCTAGTGCATTGTCATAGGA-30) which amplify a 100 bp fragment; the probe: (50-[6 FAM] TCC GACCGCGCTCCGACCGACG-[TAMRA-Q]-30), was synthesized and conjugated with the reporter dye FAM and TAMRA quencer dye, which were covalently linked to 50 and 30 ends oligonucleotide respectively. The reaction was optimized to obtain the best amplification kinetics and the cycle condition was performed for 1 cycle, 15 min at 95 °C, 30 s at 95 °C and 50 s at 60 °C for 50 cycles^77^. Corbett Instrument and Rotor-Gene 6000 Series Software 1.7 (Build 87) were used for PCR and analysis (Corbett Research, a Division of Corbett Life Science, ISO 9001:2000, reg. No. QEC21313).

### Isolation of placental mononuclear cells (PlMC)

Pieces of term placenta (10 g) were thoroughly washed in sterile phosphate buffered saline (PBS) until PBS became clear. Single cell suspensions were prepared by mechanical disruption of tissue (5 g) in sterile PBS followed by sequential filtrations through 100 µm metal sieve and 60 µm strainer (Becton Diskinson) and centrifugation at 1500 rpm for 15 min. The pellet was resuspended in 4 ml sterile PBS, layered on 2 ml Histopaque (Sigma Aldrich), and spun at 800 × g for 20 min (without break). The mononuclear cells were removed from the interface, washed, and resuspended at 1 × 10 e6 cells/ml in PBS/0.1% bovine serum albumin (BSA).

### Cell labeling and FACS

For determination of CD3-, γδ- and Vδ2-cell numbers by FACS, PlMC were stained using the following antibodies: CD3-FITC (clone UCHT1, Immunotools), TCR γδ-PE (clone F11; BD), Vd2-PerCP (clone B6, Biolegend). Three color flow cytometry was performed using a FACSCallibur instrument (BD Biosciences, San Jose, CA), compensated with single fluorochromes and supported with CellQuestPro as acquisition and data analysis software (BD Biosciences). Isotype-matched immunoglobulins were used as controls for nonspecific immunofluorescence. Dead cells were excluded utilizing appropriate forward and side scatter selection on flow cytometry. The lymphocytes were gated using forward/sideward scatter gating. CD3 cells were analyzed within the lymphocyte gate, TCR γδ cells - within CD3+ T-cell gate and Vδ2 cells – within γδ+ T-cell gate.

### Statistical analysis

For statistical analyzes GraphPad Prism v.4 (San Diego, CA) was used. To quantitate changes in the numbers of CD3, γδ and Vδ2 cells between placenta samples with and without mycobacterial L-forms Student t test was performed. The p values < 0.05 were considered to reflect significant differences.
